# Chondrogenic and Gliogenic Subpopulations of Neural Crest Play Distinct Roles during the Assembly of Epibranchial Ganglia

**DOI:** 10.1371/journal.pone.0024443

**Published:** 2011-09-09

**Authors:** Maya D. Culbertson, Zachary R. Lewis, Alexei V. Nechiporuk

**Affiliations:** Department of Cell and Developmental Biology, Oregon Health & Science University, Portland, Oregon, United States of America; Texas A&M University, United States of America

## Abstract

In vertebrates, the sensory neurons of the epibranchial (EB) ganglia transmit somatosensory signals from the periphery to the CNS. These ganglia are formed during embryogenesis by the convergence and condensation of two distinct populations of precursors: placode-derived neuroblasts and neural crest- (NC) derived glial precursors. In addition to the gliogenic crest, chondrogenic NC migrates into the pharyngeal arches, which lie in close proximity to the EB placodes and ganglia. Here, we examine the respective roles of these two distinct NC-derived populations during development of the EB ganglia using zebrafish morphant and mutants that lack one or both of these NC populations. Our analyses of mutant and morphant zebrafish that exhibit deficiencies in chondrogenic NC at early stages reveal a distinct requirement for this NC subpopulation during early EB ganglion assembly and segmentation. Furthermore, restoration of wildtype chondrogenic NC in one of these mutants, *prdm1a*, is sufficient to restore ganglion formation, indicating a specific requirement of the chondrogenic NC for EB ganglia assembly. By contrast, analysis of the *sox10* mutant, which lacks gliogenic NC, reveals that the initial assembly of ganglia is not affected. However, during later stages of development, EB ganglia are dispersed in the *sox10* mutant, suggesting that glia are required to maintain normal EB ganglion morphology. These results highlight novel roles for two subpopulations of NC cells in the formation and maintenance of EB ganglia: chondrogenic NC promotes the early-stage formation of the developing EB ganglia while glial NC is required for the late-stage maintenance of ganglion morphology.

## Introduction

In the developing vertebrate embryo, neurons of the facial, glossopharyngeal and vagal sensory ganglia are derived from transient thickenings of the embryonic ectoderm known as the epibranchial (EB) placodes. These placodes comprise regions of simple columnar epithelium that form dorsal to the developing branchial arches during the early stages of somitogenesis. Ganglion development commences shortly after the end of somitogenesis as neuroblasts delaminate from the EB placodes and move medially. Here they converge with a subpopulation of cranial neural crest (NC) which differentiate into the glial cells of the EB ganglia [1]. In addition to the gliogenic NC, the chondrogenic NC precursor subpopulation also comes into close proximity with the forming EB ganglia. The gliogenic and chondrogenic NC precursors migrate together with pigment precursors in three streams from the rostral neural tube to the ventral head prior to the onset of EB gangliogenesis [2], [3]. While the first, anterior-most stream migrates rostral to the developing EB ganglia, the second and third stream of NC respectively give rise to the hyoid bones and five ceratobranchial arches of the ventral head skeleton in addition to the EB glia [3].

The proximity of NC- and neurogenic placode-derived cells in the vertebrate head is suggestive of signaling interactions between the two populations. However, previous studies on the nature of these interactions have yielded somewhat conflicting results. In some cases, ablation of NC led to failures in ganglion formation. Excising chick neural folds to remove NC in one report resulted in aberrant trigeminal ganglion condensation even though placodal cells retained the ability to ingress and proceed with neurogenesis [4]. A subsequent study suggested that a Slit1-Robo2 signaling pathway might mediate similar interactions between the NC and EB placode-derived neuroblasts during EB ganglia assembly [5]. Yet a third study in chick yielded different results: neuronal markers were upregulated in EB placode cells following extirpation of the NC, suggesting that the EB placodal precursors were still able to initiate neurogenesis in the absence of NC. However, the normal delamination and ingression of EB placode cells failed to occur, resulting in abnormally superficial placement of nascent neurons [1]. Interestingly, another study in mouse found that the facial ganglion forms normally following specific ablation of the NC via *Wnt1::Cre*-driven expression of diphtheria toxin, suggesting that ganglion formation could proceed independent of NC [6]. Thus, further studies are needed to address what role, if any, the NC plays during EB ganglia formation in vertebrates.

Though removal of NC in different contexts has yielded disparate results, it is worth noting that the NC is a heterogeneous population and that simultaneous ablation of gliogenic and chondrogenic NC may mask any differential effects that NC subpopulations may have on placode development. Moreover, if placodal cells are only competent to respond to signals from specific NC derivatives at certain stages, then removing the NC at various developmental stages may have different consequences for ganglion assembly. We therefore employed zebrafish models with known alterations in different NC populations to address whether gliogenic or chondrogenic NC affect ganglion assembly and neurogenesis.

A number of previously characterized mutations affect the prevalence of either chondrogenic or glial subtypes derived from NC. *Sym1*, a hypomorphic mutation in *foxd3* is characterized by marked reductions in early-stage NC marker expression and subsequent losses in both glial precursors and ventral head skeleton structures derived from the anterior chondrogenic NC streams [7]. In contrast, the *colourless (cls)* zebrafish mutant contains a lesion in the SRY-related HMG box transcription factor *sox10* and exhibits an early loss of non-ectomesenchymal NC derivatives (i.e., melanocytes and glia) yet displays normal development in ectomesenchyme-derived elements such as ventral head skeleton and medial fin folds [8]. Antisense morpholino-mediated disruption of another factor, Disc1, promotes the adoption of glial fates at the expense of chondrogenic fates via regulation of *foxd3* and *sox10*, which implies an early-stage change in NC precursor cell fate decision that well-precedes the formation of the cranial ganglia [9]. Finally, *narrowminded (nrd)*, a putative null truncation mutant in *prdm1a* (also referred to as PRDI-BF or *blimp-1*), exhibits defects in NC-derivatives such as fin fold mesenchyme and melanophores [10] with dysmorphic changes in the ventral head skeleton [11]. These disruptions are consistent with early-stage reductions in multiple NC lineages that are observable well before the onset of cranial gangliogenesis [12].

In zebrafish, the transition from placode induction to assembly of cranial ganglia from neurogenic precursors can be tracked through the course of development by visualizing distinct markers at progressive stages. Early-stage placode specification and induction is accompanied by upregulation of the transcription factor Pax2a (11–24 hours post-fertilization – hpf) [13]. Subsequent neurogenesis in placode-derived precursors is marked by upregulation of two transcription factors: *phox2b* is specifically expressed in migrating neuroblasts and the forming ganglia beyond 30 hpf, while the neural marker Elavl3/4 (previously called Hu-C/D) is expressed in mature neurons and thus marks the EB ganglia during later stages (72–96 hpf) [14], [15].

In the present study, we examine the development of cranial ganglia in various models of NC depletion and find disparate roles for two NC subtypes, glial and chondrogenic, during EB gangliogenesis. We demonstrate that chondrogenic, but not glial derivatives of the NC are required for the nascent formation of EB ganglia. Moreover, transplantation analyses reveal that the chondrogenic NC is necessary and sufficient for restoring EB ganglion formation in a mutant background. We also identify a discrete role for glial NC derivatives in determining the late-stage morphology of the EB ganglia.

## Results and Discussion

### Neural Crest Cells Migrate to Abut Placodal Ganglionic Neural Precursors

**Figure 1:**
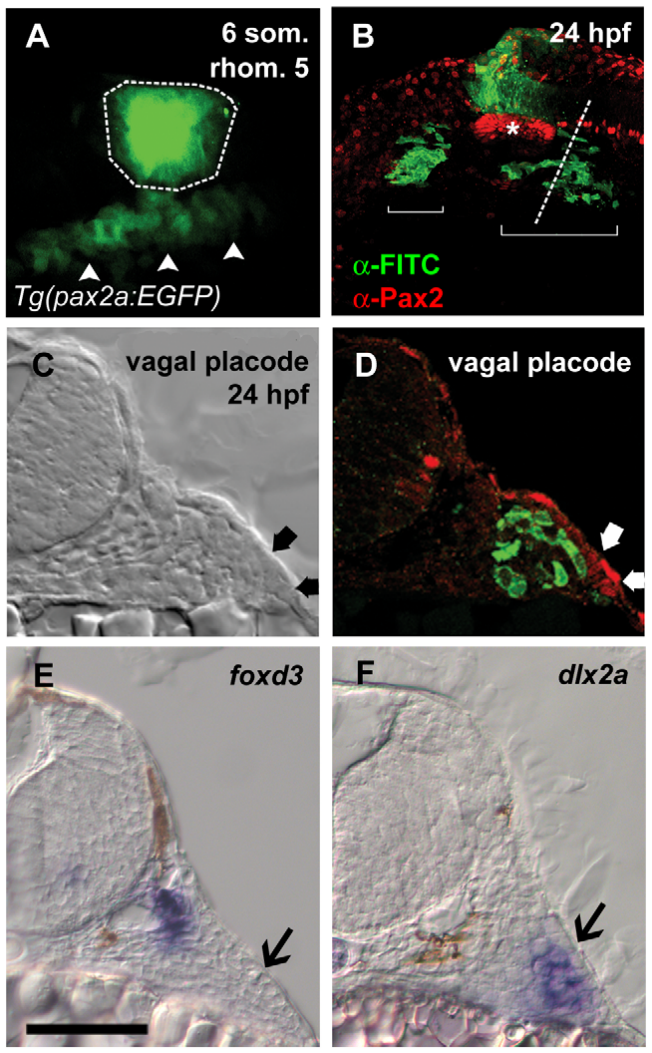
Neural crest cells migrate to abut epibranchial placodes. (**A**) Lateral view of *Tg(pax2a:EGFP)^w37/+^* embryo at the six somite stage showing fluorescein uncaged in rhombomere 5 (outlined region). Placode precursor field expressing EGFP is visible below the uncaged region (arrowheads). (**B**) Lateral view of the same embryo at 24 hpf. Fluorescein-labeled NC cells derived from rhombomere 5 (green) are visible in two regions (brackets) ventral to the otic vesicle (asterisk). (**C** and **D**) Transverse section through embryo at level indicated by dotted line in B. Placode is visible as thickened ectoderm under transmitted light and by α-Pax2 staining in red (C and D, thick arrows). In D, fluorescein-labeled cells are just medial to, but excluded from, the placode. (**E** and **F**) *In situ* hybridization at the level of the vagal placode shows neural crest-derived glial precursors adjacent to the neural tube expressing *foxd3*; in F, a group of cells corresponding to the fluorescein-labeled region in D expressing the chondrogenic crest marker *dlx2a*. Placodes in E and F indicated by black arrows. Scale bar = 50 µm.

In order to determine the spatial and temporal relationship between the cranial NC and the EB placodes and ganglia, we visualized these cell populations in both live and fixed samples during early development. We began by employing time-lapse analysis to determine the relationship between the migratory NC and the forming EB ganglia. Using photoconvertible Kaede protein, we marked premigratory NC cells at 16 hours post-fertilization (hpf) and tracked their movement through 30 hpf [16]. These embryos also carried the *TgBAC(neuroD:EGFP)^nl1^* transgene, which marked developing cranial ganglia [17]. We observed that by 18 hpf, NC cells derived from rhombomere 6 populated the area ventral to the forming posterior lateral line ganglion (Movie S1). The EGFP-positive cells, which mark the condensing vagal ganglion, appeared in this region by 28 hpf; well after the arrival of the NC. To corroborate this observation and clarify the three-dimensional relationship between post-migratory NC cells and EB placodes, we injected caged fluorescein dextran into zygotes and uncaged a region corresponding to rhombomere 5 (r5) prior to the onset of NC migration (12 hpf; Fig. 1A). As expected, by 24 hpf, NC cells derived from the r5 level contributed to the second and third cranial streams, situated anterior and posterior to the otic vesicle, respectively. Importantly, a subset of labeled NC cells appeared immediately adjacent to the respective anterior and posterior positions of the presumptive facial and combined glossopharyngeal/vagal placode fields (Fig. 1B). Transverse sections through the posterior aspect of the vagal placode (Fig. 1C,D) revealed that the NC cells were situated immediately adjacent to, but excluded from, the placode itself. We conclude that NC cells flank the placodal ectoderm prior to the onset of neurogenesis.

To define the identity of cells populating the regions dorsal and ventral to the placodes, we used *in situ* hybridization for NC-specific markers. *foxd3*, which labels post-migratory NC derived glia, was expressed in the area immediately lateral to the neural tube, consistent with the location of postmigratory glial precursors (Fig. 1E). *dlx2*, which is a specific marker of post-migratory chondrogenic NC situated in the branchial arches, was expressed adjacent to the placode but in a more ventral location than *foxd3* (Fig. 1F). Close proximity between glial and chondrogenic NC derivatives and EB placodes suggested possible interactions between these populations during the process of cranial ganglion assembly. We therefore examined mutants and morphants in which these two NC populations were altered.

### Loss of Chondrogenic Neural Crest Results in Various Ventral Head Skeleton Defects

**Figure 2:**
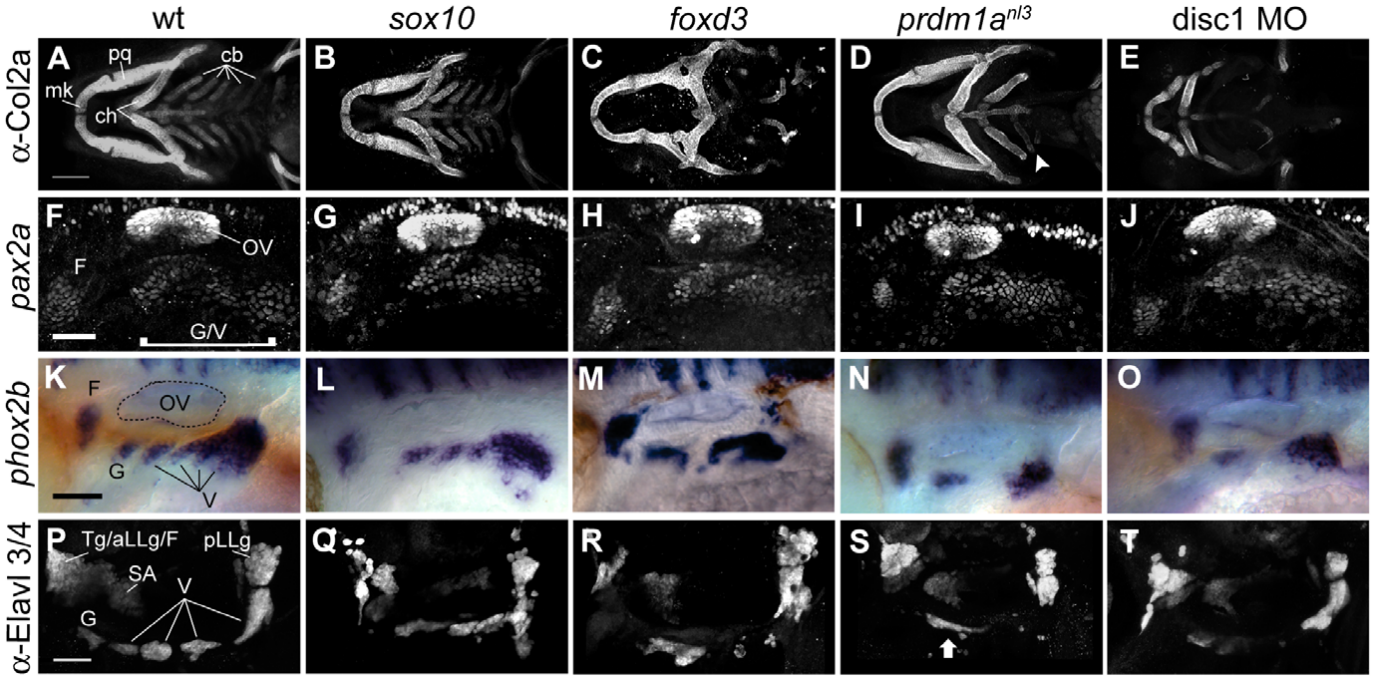
Disruptions in neural crest cause late defects in epibranchial ganglion morphology without affecting placode specification. (**A–E**) Ventral view of head skeleton visualized with anti-Collagen2a antibody at 4 dpf. (A) The following structures are visible in wildtype: Meckel's cartilage, palatoquadrate, ceratohyal and ceratobranchial arches. *Sox10* mutant (B) exhibits normal morphology. Radical disruption of structure is apparent in *foxd3* mutants (C) and *disc1* morphants (E). *prdm1a* mutant (D) displays grossly normal anterior jaw structures and deficits in the posterior arches; in this example, an unpaired ceratobranchial vestige can be seen (arrowhead). (**F–J**) Anti-Pax2 immunofluorescence at 24 hpf. Wildtype (F) shows facial placode and unsegmented glossopharyngeal/vagal placode field (**K–O**) *phox2b* expression at 48 hpf. Wildtype (K) displays normal segregation of facial,glossopharyngeal, and vagal ganglia. Defects are apparent in *foxd3* (M) and *prdm1a* (N) mutants and *disc1* morphant (O). *sox10* mutant (K) display morphology and segmentation comparable to wildtype siblings (F). **P–T:** Ganglion morphology visualized by anti-Elavl3/4 antibody staining at 4 dpf. (K) Wildtype control shows glossopharyngeal and vagal ganglia. Posterior lateral line, statoacoustic and the fused trigeminal, anterior lateral line and facial ganglia can also be seen. Defects are apparent in *sox10* (Q), *foxd3* (R), *prdm1a* (S) mutants and *disc1* morphant (T). Arrow in (S) shows an example of fusion often seen in *prdm1a* mutants. Abbreviations: mk = Meckel's cartilage; pq = palatoquadrate; ch = ceratohyal; cb = ceratobranchial arches; OV = otic vesicle; F = facial ganglion; Tg/aLL/F = trigeminal/anterior lateral line/facial ganglion complex; SA = statoacoustic ganglion; pLLg = posterior lateral line ganglion; G = glossopharyngeal ganglion; V = vagal ganglia. Scale bar in (A) = 100 µm. Scale bars in (F), (K) and (P) = 50 µm.

To elucidate NC influences on the formation of EB placodes and ganglia, we examined the formation of these structures in the s*ox10*, *foxd3* and *prdm1a^nl3^* mutants and the *disc1* morphant, all of which exhibited deficiencies in chondrogenic and/or glial NC. Since the cartilaginous elements of the developing mandible and pharyngeal skeleton segment according to the three anterior streams of NC from which they arise, we posited that the structure of the ventral head skeleton could be used as a readout for early-stage disruption of individual chondrogenic NC streams. We therefore examined the jaw skeleton via immunohistochemical staining for Collagen2a at 4 days post-fertilization (dpf) in these mutant and morphant conditions (Fig. 2A–E). Previous studies have also indicated that both *sox10* and *foxd3* mutants have a significant reduction or absence of the NC-derived cranial glia [8], [18]. The *sox10* mutant showed no discernable defects in skeletal morphology or articulation (Fig. 2B), confirming prior assertions that the chondrogenic NC is undisturbed [8]. The *foxd3* mutant, on the other hand, exhibited a generalized jaw phenotype; though it was variable, it most often resulted in foreshortened and malformed anterior mandibular and hyoid bones with missing and/or vestigial pharyngeal arches (Fig. 2C). This phenotype was consistent with previously published reports of chondrogenic NC reduction associated with the loss of Foxd3 [7], [18], [19].

To analyze craniofacial structure in the absence of Prdm1a activity, we utilized a mutant isolated during the course of an ongoing forward genetic screen, *prdm1a^nl3^* (see Methods section for details). *prdm1a^nl3^* showed preservation of anterior and medial jaw structures, with grossly normal morphology of Meckel's cartilage, the palatoquadrate and hyoid bones; this suggests that the first and second NC streams are largely intact. The ceratohyal arches, on the other hand, were severely disrupted; only the most anterior pair of arch bones formed normally while more posterior arches did not (Fig. 2D). The bones of the second ceratobranchial arch were sometimes, but not always, formed and were usually smaller than expected and often unpaired, if present at all (Fig. 2D, arrowhead). This suggests that chondrogenic NC defects in *prdm1a^nl3^* mutants are limited to the third stream. However, the distribution of gliogenic cranial NC derivatives in *prdm1a^nl3^*, as visualized by *foxd3* expression at 48 hpf (Fig. S1), was indistinguishable from that seen in wildtype controls; this demonstrates that chondrogenic NC loss was not accompanied by glial NC loss. Finally, *Disc1* morphants exhibited the most severe skeletal defects. Previous work indicated that the reduction in the chondrogenic NC was accompanied by a concomitant expansion of the cranial glia [9]. Consistent with this result, the *Disc1* morphant presented with a complete loss of pharyngeal arches and severely malformed anterior jaw structures, suggesting that the dysfunction encompasses all anterior chondrogenic NC streams (Fig. 2E). With a series of mutants at our disposal that differentially altered glial and chondrogenic NC populations to varying degrees, we proceeded to examine how NC subtypes affected the development of the cranial ganglia.

### Disruptions in Neural Crest Cause Defects in Epibranchial Ganglion Morphology Without Affecting Placode Specification

Having confirmed the nature of chondrogenic NC disruptions in our mutant and morphant lines, we then set out to determine if defects in NC alter the initial specification of the EB placodes. In wildtype embryos, labeling with an α-Pax2 antibody at 24 hpf marked the developing otic vesicle (OV) as well as the facial placode and the combined glossopharyngeal and vagal placode domain (Fig. 2F). Though we were unable to determine whether both Pax2 paralogues, Pax2a and Pax2b, were recognized by this antibody, the protein distribution it marked mirrored the expression pattern obtained with *in situ* hybridization for *pax2a*: a factor known to be upregulated during placode induction [13]. This wildtype staining pattern was preserved in the s*ox10*, *foxd3* and *prdm1a* mutants subpopulations., and *disc1* morphant (Fig. 2G–J). As Pax2a expression is not altered in any of our NC depletion models, we can conclude that EB placode specification progresses irrespective of the presence or absence of glial or chondrogenic NC.

We next tested the requirement of NC subpopulations in the condensation of the EB ganglia by examining 48 hpf embryos for the expression of *phox2b*, which is upregulated in nascent EB ganglia during early neurogenesis. (Fig. 2,K–O). *sox10* mutants, whose glia were reduced without a concomitant loss of chondrogenic NC precursors, exhibited normal early condensation and segmentation of the epibranchial ganglia using this marker (Fig. 2L). By contrast, mutants whose chondrogenic NC precursors were altered exhibited defects in ganglion structure. The *foxd3* mutant displayed marked deformity in the ganglia (Fig. 2M) while the *prdm1a* mutant and *disc1* morphant (Fig. 2N, O) evidenced decreased *phox2b* expression in addition to discernable alterations of ganglionic structure. In order to confirm that ganglion dysmorphosis was attributable to disturbances in NC populations, we examined other known prerequisites for EB gangliogenesis: specifically, the presence of endodermal pouches and cell survival. Because signals derived from the endodermal pouches are necessary for EB placode neurogenesis [14], [15], we examined pouch integrity in the mutants and *disc1* morphant. *In situ* hybridization for *nkx2.3* at 2 dpf or fluorescent immunostaining against Zn5 at 2 dpf revealed grossly normal endodermal pouch morphology in all cases (Fig. S2) indicating that failures in ganglion assembly could not be attributed to the absence of endodermal signaling.

We then proceeded to determine whether EB placode-derived precursors were succumbing to cell death, as a loss of neuroblasts could also result in improper EB ganglion formation. We therefore processed the *foxd3*, *sox10* and *prdm1a^nl3^* mutants and *disc1* morphants for TUNEL labeling at 30 hpf and examined the region of the EB placodes. In all cases, the number of TUNEL-reactive cells was not significantly different from wildtype controls, indicating that failures in EB ganglion assembly was not attributable to increased cell death (data not shown). Having established that neuroblast precursors were not being lost to cell death, we then asked whether a relative decrease in neurogenic progenitor proliferation could account for the loss of ganglia in glia- and chondrocyte-deficient mutants. To test this, we performed simultaneous immunostaining for Phospho-histone H3 (to mark dividing cells) and Pax2 (to label EB placodes) in glia-depleted *sox10* and chondrocyte-depleted *prdm1a^nl3^* mutants and their respective wildtype siblings at 30 hpf. There was no significant difference in mitotic rates between mutants and wildtypes for either *sox10* (mutant mean of 0.0415+/−0.0084 versus wildtype mean of 0.0578+/−0.0084; *p*<0.28) or *prdm1a^nl^* (mutant mean of 0.0275+/−0.0077 versus wildtype mean of 0.0286+/−0.0169; *p*<0.44). From this, we concluded that EB ganglion disruption was not the result of decreased cell proliferation.

In order to observe how the loss of chondrogenic NC affects ganglion assembly at early stages, we employed timelapse imaging to visualize the dynamics of neuroblast cell migration and ganglion condensation in wildtype and *prdm1a^nl3^* mutants. Wildtype and mutant embryos carrying the *TgBAC(neuroD:EGFP)^nl1^* transgene were imaged in the region around the otic vesicle for 15–18 hour periods during the onset of ganglion assembly beginning at 31 hpf. In wildtype embryos (Movie S2), cells ventral to the otic vesicle begin to upregulate EGFP and move dorsally; this migration most likely corresponds to the delamination of EB placode neuroblasts (see Movie S2; arrows at 33.52 hpf mark). The cells then began to aggregate, coalescing just ventral to the posterior lateral line ganglion that corresponds to the nascent major vagal ganglion. By 45 hpf, the minor vagal ganglia proceeded to segment along the ventral edge of the fused vagal ganglion body (see Movie S2; labels at 45.84 hpf mark). In the *prdm1a^nl3^* mutant (Movie S3), EB placode neuroblasts upregulated EGFP and migrated dorsally and began to coalesce as in the wildtype; however, during the initial assembly of the major vagal ganglion fewer cells were seen emanating from the placodal field. Vagal ganglion segmentation also failed to occur; the large vagal ganglion appeared elongated and showed no signs of segmenting into smaller ganglia. In addition, a small group of cells unassociated with a ganglionic cluster were observed adjacent to the ventral edge of the otic vesicle (Movie S3; 46.40 hpf mark, asterisk). These data collectively indicate that ganglion deformation seen in *prdm1a^nl3^* embryos stems from dysfunctions during initial assembly. Taken in context with the alterations in EB ganglion formation in the mutants and morphant, we conclude that chondrogenic derivatives of NC are necessary for the correct assembly and segregation of the ganglia at early stages.

Next we asked whether the positioning and differentiation of the EB ganglion neurons were affected by the absence of the chondrogenic and/or glial NC during later neurogenic stages. Immunolabeling against the neuronal marker Elavl3/4 at 4 dpf (Fig. 2P–T) revealed missing minor vagal ganglia and malformation of the large vagal ganglion in the *foxd3* mutant and *disc1* morphant (Figs. 2R and T). The *prdm1a* mutants also exhibited incorrect segmentation and/or absence of glossopharyngeal and small vagal ganglia; this was often accompanied by a cluster of immunoreactive cells in the region associated with the first branchial arch (Fig. S2, arrow). The late-stage defects in ganglion morphology in these three cases is not surprising given that dysfunctions in ganglion assembly were discernable two days earlier. By contrast, the *cls/sox10* mutant at this stage showed ganglion dymorphosis despite apparently normal development at earlier stages (Fig. 2Q). In this case, Elavl3/4-positive neurons were present in the general area of expected ganglion formation but failed to segregate into the series of discrete structures seen in the wildtype.

Since nascent ganglion formation and segmentation appear to proceed normally in embryos with deficiencies in gliogenic NC, we employed timelapse microscopy in *TgBAC(neuroD:EGFP)^nl1^* transgenic embryos to ask whether we could detect differences in late-stage ganglion morphology between *sox10* mutant larvae and their wildtype cohorts. We again imaged the region around the otic vesicle, this time beginning at 80 hpf, taking images every 15 minutes over a 17.25 hour period to capture any changes in the structure of the cranial ganglia. In the wildtype larva (Movie S4), the EB ganglia were already organized into discrete structures and displayed little discernable cell movement. The *sox10* mutant, on the other hand, exhibited ganglia that were less defined compared to those of the wildtype larva, with evidence of neuroblast dispersal in the ganglion field (Movie S5). Furthermore, small clusters of cells could be observed moving appreciably (Movie S5, arrows) as the overall appearance of the ganglia became less organized over time. This suggests that the glial component of NC, while not required during early neurogenic stages, plays a later role in determining cranial ganglion architecture.

### Disruption of Gangliogenesis is Closely Correlated With Loss of Ceratobranchial Arches

Although aberrant EB ganglion formation occurs in cases of chondrogenic NC depletion, it was unclear whether EB ganglion loss is caused by segmental NC precursor losses, or as a general consequence of depletion in NC subpopulation overall. To answer this question, we examined formation of the EB ganglia in individual *prdm1a^nl3^* mutants that exhibit variable loss of the first three ceratobranchial arches. Mutant larvae expressing *TgBAC (neuroD:EGFP)^nl1^* were immunostained with α-Collagen2a to visualize both the EB ganglia and the associated head skeleton elements (n = 11; Fig. S3). We observed that individual ganglia tended to form when the associated ceratobranchial arch was present. In cases where only the first ceratobranchial arch formed, the glosspharyngeal ganglion condensed, though the small vagal ganglia did not. However, if a dysmorphic second ceratobranchial arch formed in addition to the first, a cluster of EGFP-positive cells would condense in what likely corresponds to a fusion body of the glossopharyngeal and first vagal ganglia (Fig. S3B, C). In rare cases where the second ceratobranchial arch formed normally, the glossopharyngeal and first vagal ganglia were present as discrete structures (Fig. S3D). In a single case, a group of Collagen2-reactive cells resembling a vestige of the third ceratobranchial arch was associated with a small, EGFP-positive cell cluster positioned just ventral to the expected location of the second vagal ganglion (Fig. S3E).

Further examination of the second ceratobranchial arch in *prdm1a^nl3^* mutants confirmed close association between the presence of this skeletal structure and the associated ganglion (Table S1). We concentrated on this arch because the frequency of its formation exhibits the most variability in this mutant. In 7 of the 11 embryos examined, the second ceratobranchial arch was completely absent and of these 7, only one exhibited an identifiable first vagal ganglion. In 3 cases, a dysmorphic second ceratobranchial arch was present. Here, only 2 first vagal ganglia formed: one being dysmorphic and the second as part of a fusion body with the presumptive glossopharyngeal ganglion. In one case, the second ceratobranchial arch formed normally and again, the first vagal ganglia formed within a fusion body. All together, this indicate that the loss of individual EB ganglia is highly consistent with ceratobranchial arch loss.

### Restoration of the Chondrogenic But Not Glial Neural Crest in *prmd1a* Mutant is Sufficient to Rescue EB Ganglia Condensation

**Figure 3:**
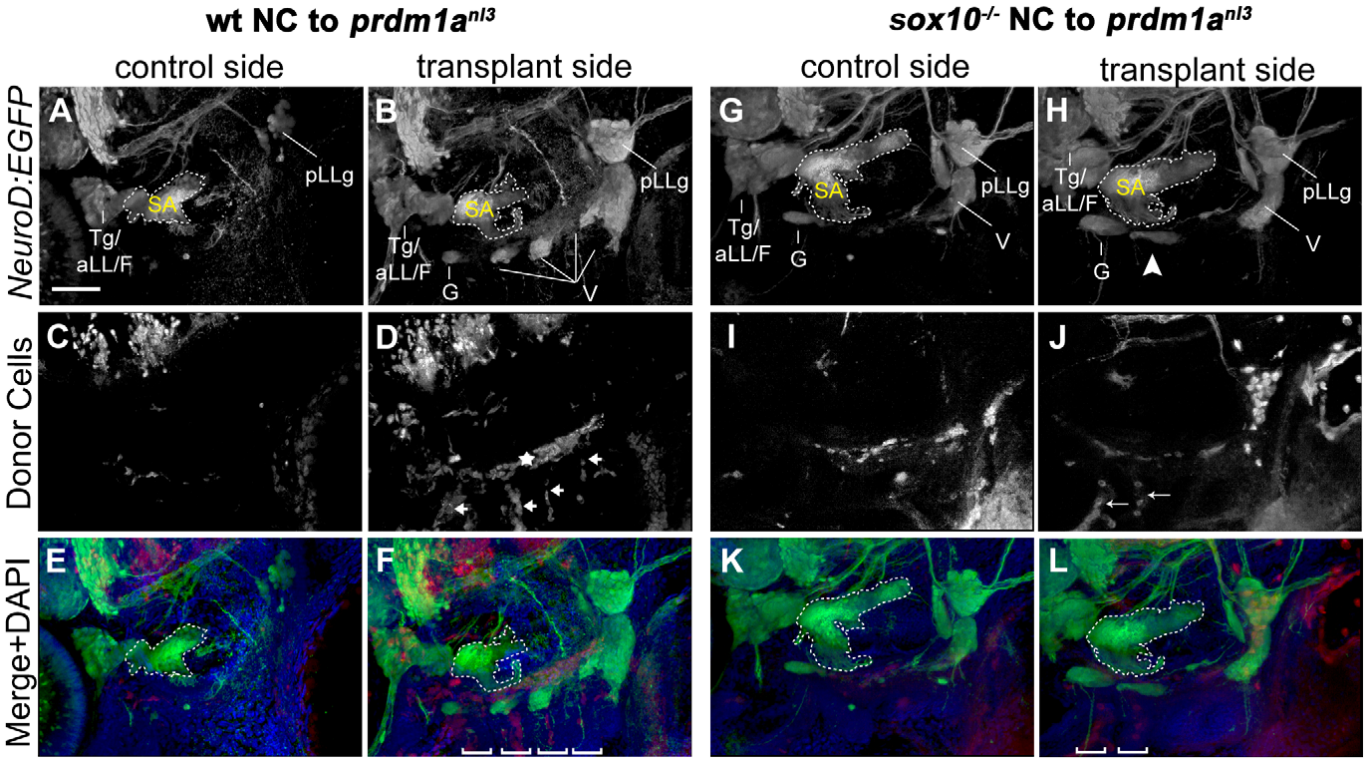
Wildtype and *sox10^−/−^* NC is capable of rescuing cranial ganglion formation in *prdm1a^nl3^* mutant embryos. (**A–F**) EB ganglion rescue mediated by transplantation of wildtype NC into *prdm1a^nl3^* mutant embryos expressing T*g(NeuroD:EGFP)*. (**A, B**) Left (control) and right (transplanted) lateral views of *prdm1a^nl3^* mutant head with rhodamine-positive wildtype NC donor cells at 3 dpf. *Tg(neuroD:EGFP)^nl1^* expression reveals an absence of identifiable cranial ganglion structures on the non-transplanted side (A) while transplanted side shows restoration of these structures, including identifiable glossopharyngeal and vagal ganglia. (**C, D**) Distribution of rhodamine-positive donor NC cells shows an absence of fluorescent label in the cranial ganglion field of the non-transplanted side (C). In the transplant side (D), rhodamine-positive donor cells are present in parallel columns extending ventrally from the ganglion field (arrows) and in a single line perpendicular and immediately dorsal to columns (star), likely corresponding to the ceratobranchials and the rostral basicranial commisure, respectively. (**E and F**) Merged channel high-magnification views of control (E) and rescue (F) sides with DAPI counterstain (blue). Note DAPI-labeled branchial arches containing rhodamine-positive donor cells (brackets). (**G–L**) Partial EB ganglion rescue following transplantation of *sox10^−/−^* NC cells into *prdm1a^nl3^* host embryos. (**G,H**) *Tg(NeuroD:EGFP)* expression in *prdm1a^nl3^* embryo showing absence of small EB ganglia in control side (G) and a partial rescue of ganglion formation in ventral vagal region of transplanted side (arrowhead). (**I,J**) Distribution of rhodamine-labeled donor cells in control side (I) and rescue side (J). (**K,L**) Merged channel high-magnification views of control (K) and rescue (L) sides with DAPI counterstain. Rescued side shows juxtaposition of rescued ganglion with the rhodamine-positive arches (brackets). Margins of statoacoustic ganglia are indicated by dashed white lines. Abbreviations: Tg/aLL/F = trigeminal/anterior lateral line/facial ganglion complex; SA = statoacoustic ganglion; pLLg = posterior lateral line ganglion; G = glossopharyngeal ganglion; V = vagal ganglia. Scale bar in (A) = 50 µm.

Because loss of chondrogenic NC and their derivative structures in the *prdm1a^nl3^* mutant had demonstrable effects on the morphology of the EB ganglia, we asked whether restoration of the branchial arches in this mutant was sufficient to rescue ganglion condensation. We performed unilateral transplants of rhodamine-labeled cells from blastulae-staged wildtype donor into the presumptive NC fields of gastrula-staged *prdm1a^nl3^* hosts and assayed the cranial ganglia in the chimeric embryos using *TgBAC(neuroD:EGFP)^nl1^* transgene expression. Because transplants were performed prior to the six-somite stage, which is when cranial NC cells were previously described crossing the dorsal midline [9], some transplanted NC cells localized contralateral to the targeted side, though this subset generally comprised a smaller proportion when compared to the targeted side. When wildtype NC was transplanted into *prdm1a^nl^* mutant embryos, one or more cranial ganglia formed in the side of the embryos that received wildtype NC (Fig. 3, A–F) in 16 cases (15%; n = 108 total *prdm1a^nl3^* hosts) whereas rescue was never observed in the control side. In those instances where rescue was observed, rhodamine-positive wildtype donor cells lined up dorsoventrally within rescued ceratobranchial arches (Fig. 3D, arrows). These columns of wildtype NC cells corresponded to the pattern of Collagen II antibody staining in the ceratobranchial arches as seen in lateral view of the larval ear at 5 dpf further indicating that the donor cells were NC-derived chondrocytes (Fig. S4A). In a number of cases, donor cells also populated a linear domain located superior to the glossopharyngeal and minor vagal ganglia analogous to the rostral basicranial commissure (RBC) in the 5 dpf larva (Fig. S4A, asterisk). It is worth noting that the RBC forms normally in *prdm1a^nl3^* mutants and is therefore unlikely to be the mediator of cranial ganglion restoration in rescued larvae.

The configuration of donor cells in cases of rescue, while similar to the structure of chondrogenic NC in older larvae, does not resemble the distribution of glia proximal to the ganglia as visualized at 5 dpf with an antibody against the myelin marker MBP (Fig. S4B). This suggests that rescue occurs when donor cells adopt NC fates that are predominantly chondrogenic rather than glial. Importantly, when columnar distribution of transplanted NC and morphology via DAPI labeling indicated complete rescue of branchial arch morphology, we always observed rescue of the overlying cranial ganglion. Conversely, we never observed restoration of branchial arches incorporating transplanted cells without concomitant ganglion rescue. As restoring NC is able to rescue cranial ganglion development in these genetically-deficient hosts, we conclude that EB ganglion loss in the *prdm1a^nl3^* mutant is a specific consequence of branchial arch ablation rather than a direct effect of Prdm1a on gangliogenic processes or other tissues.

To confirm that it was the chondrogenic, and not the glial, component of NC that mediated rescue of EB ganglion formation, we undertook another set of transplant experiments, this time applying *sox10* mutant NC to *prdm1a^nl3^* mutant hosts. We reasoned that as the *sox10* mutant NC is depleted of glial derivatives, any recovery of ganglion structures seen in these cases would be attributable to chondrogenic NC restoration. We found that the NC cells derived from *sox10* donors were able to rescue ganglion structures at about the same frequency as the NC derived from wildtype donors (17% or 4 out of 23 total vs. 15% rescue for wildtype donors; Fig. 3, G–L). These results led us to conclude that chondrogenic, but not glial NC, is specifically required for the initial condensation of the EB ganglia.

Prior approaches to discerning the role of the NC in cranial ganglion formation have not specifically addressed the functions of those distinct subpopulations that comprise the NC as a whole. While complete ablation of the NC has been shown in previous studies to be deleterious to the development of cranial sensory ganglia, the respective roles of individual NC components in directing proper ganglion development remained unclear. By taking the heterogeneous and pluripotent nature of the NC into account, we were able to gain insight into the effects of NC disruption by employing gene-mediated reductions of specific subpopulations and observing the effects on EB ganglion development over time. Our findings reveal stage- and population-dependent requirements for the NC during the development of cranial sensory ganglia. We demonstrate that the NC employs two different aspects of its cellular population, chondrogenic and gliogenic precursors, at specific stages to facilitate both the initial formation and late-stage structural refinement and maintenance of the cranial sensory ganglia. Specifically, we find that the chondrogenic NC, but not its gliogenic counterpart, is indispensible for the initial condensation of the cranial ganglia. By contrast, the glial component is required to maintain ganglion integrity following condensation. In conclusion, our work has uncovered discrete, previously unappreciated roles for individual subpopulations of NC in cranial ganglion development.

Our results may serve to bridge the gap between some of the disparate results yielded by previous chick and mouse studies. Extirpation of the NC in chick prevented normal delamination and ingression of EB placodal cells [1]. In line with this result, we failed to observe ganglionic assembly of placode-derived neuroblasts in cases where NC-derived skeletal structures were lost. These results apparently conflict with a 2008 study by Shiau and Bronner-Fraser which found that following miRNA-mediated knockdown of Slit1 in the NC, Robo2-expressing placodal precursors ingress but do not properly condense to form ganglia [4]. However, these outward differences may indicate that distinct signals from the chondrogenic NC are required for ingression and condensation. For example, yet uncharacterized NC-derived signals may induce placode-derived cells to delaminate and ingress, whereas Slit1 in the NC is necessary for Robo2-positive neuroblasts to coalesce into EB ganglia. Finally, in contrast to our study in zebrafish and studies in chick [4], [5], Coppola and others concluded that the cranial NC is not required for the formation of the EB ganglia in mouse [6]. Yet it is worth noting that their study focused on the persistence of the facial ganglion following NC ablation via *Wnt1::Cre*-driven expression of diphtheria toxin; they did not scrutinize the remaining EB ganglia, the glossopharyngeal and vagal, which comprised a focal point in our study. Moreover, the efficacy of their ablation was assayed only by the loss of Sox10-positive glial precursors; other NC derivatives, including the chondrogenic subpopulation, were not examined. Our study in zebrafish, when taken in context with prior work in the chick, highlights the necessity of the NC during the formation of EB ganglia. However, further investigation in other model systems, including mouse, is needed to determine whether this requirement is conserved across vertebrate species.

## Materials and Methods

### Zebrafish strains and identification of the *prdm1a* mutant

Adult zebrafish were maintained under prescribed conditions [20]. Embryos from *AB and WIK adults were staged according to standard protocols [21]. The EB ganglia were visualized using the *tgBAC(neurod:EGFP)^nl1^* line [17]. The *prdm1a^nl13^* mutation was identified in a three-generation N-ethyl-N-nitrosourea (ENU) mutagenesis screen [22], [23] for mutants that had reduced EGFP expression in the EB ganglia. The *prdm1a^nl13^* strain failed to complement the existing *prdm1a* mutant *narrowminded* [10]. cDNA sequencing revealed a single nucleotide alteration in *prdm1a^nl13^* that results in the substitution of tyrosine for asparagine at residue 536. This lesion falls within the first zinc-finger binding domain of the two necessary for proper DNA binding [24] and results in a mutation that recapitulates and fails to complement the *nrd* phenotype.

### Uncaged Fluorescein and Kaede Cell Tracking

For fluorescein uncaging experiments *Tg(pax2a:GFP)* embryos were injected at the 1-cell stage with 1 nL of 1.25% caged fluorescein-10 kD dextran (Invitrogen) in 0.2 M KCl with 0.167% Phenol Red. Fluorophore uncaging was performed at the six-somite stage with an FV1000 confocal microscope (Olympus) with a 60× PlanApo N (1.42 N/A) oil immersion objective using a selected ROI in the Bleach function. Embryos were mounted on coverslips in 1% Low-Melt Agarose (GenePure) in embryo medium, then immersed in embryo medium. Uncaging was accomplished in selected cells by repeated scanning (4 passes) of the ROI with a 405 nm laser at 8% power. Embryos were then fixed at the 24 hpf stage in 4% paraformaldehyde/1× PBS.

For photoconverted Kaede cell tracking, *TgBAC(NeuroD:EGFP)^nl1^* were injected with 1–2 nL of 100 ng/µl Kaede mRNA at the 1-cell stage. Using similar mounting procedures, Kaede photoconversion was performed at the 14 somite stage with an FV1000 confocal microscope (Olympus) by using a specified ROI and performing four passes with the XY repeat function and the 405 nm laser at 20% power with a 2 µs dwell time. Embryos were then recorded every 6–8 minutes using timelapse z-stack acquisition over a period of ∼15 hours.

### *In Situ* Hybridization, Immunohistochemistry, TUNEL assays and Cryostat Sectioning

*In situ* hybridization was carried out as previously described [25]. Staining was performed using antisense riboprobes for *foxd3* [26], *dlx2a* [27], *phox2b* [28] and *nkx2.3* [29]. Whole-mount and sectioned embryos were photographed through an Axio Imager Z1 compound microscope with an HRc digital camera controlled by AxioImage software (Zeiss).

Fluorescent immunolabeling was performed using a published method [30] to visualize antigens of rabbit-anti-Pax2 (1∶100, Covance), mouse-anti-FITC (1∶1000, Sigma), mouse-anti Zn5 (1∶50, Zebrafish International Resource Center), rabbit-anti-phospho-histone H3 (1∶100, Cell Signaling) and mouse-anti Elavl3/4 (sold as anti-HuC/D, 1∶250, Invitrogen). For simultaneous visualization of phosopho-histone H3 and Pax2, antibody incubations were performed sequentially with an antigen masking step using a goat-anti-mouse HRP conjugated secondary antibody (1∶1000, Jackson ImmunoResearch) against anti-phospho-histone H3 prior to visualization with an anti-mouse fluorophore-conjugated antibody and subsequent staining for Pax2. Antibody staining with mouse-anti Collagen-IIa (1∶100, Iowa Hybridoma Bank) was performed using a previously described protocol with the following modifications: addition of an overnight H_2_O premeabilization wash, a 2 hour 0.05% trypsin digestion at 37°C, 2 hour 5 mg/mL hyaluronidase digestion, and use of 10% goat serum for blocking and primary antibody incubation solutions [31]. Antibody stains were completed using the applicable AlexaFluor 488 and 568-conjugated (1∶1000, Invitrogen) or Cy5-conjugated (1∶1000, Jackson ImmunoResearch) secondary antibodies and DAPI counterstaining (Sigma). TUNEL staining was performed using a TMR-red *in situ* cell death detection kit (Roche) per previously-described methods [14].

Sectioning of embryos post-staining proceeded with cryoprotection of tissue in 30% sucrose/PBS at 4°C. Embryos were then incubated at 70°C and brought stepwise through 1%, 3%, 5%, 7%, 9% and 10% concentrations of USP 100 Bloom gelatin (MP Biomedicals) in 30% sucrose/1× PBS. Embryos were embedded in 10% gelatin and the blocks fixed overnight at 4°C in 4% paraformaldehyde/1× PBS, followed by equilibration in 30% sucrose/1× PBS. Blocks were cut into 20 µm sections using a CM1850 cryostat (Leica). Sections were direct-mounted on unsubbed charged glass slides (Fisher) and coverslipped under Aquamount AQ mounting medium (Vector).

### Time-Lapse Microscopy

When applicable, mutant and wildtype siblings were treated with 0.2 mM N-phenylthiourea (i.e., PTU; Sigma) beginning at 24 hpf to prevent pigment formation. Larvae were allowed to develop to the appropriate stage and were then sedated in embryo medium containing 0.0016% ethyl-3-aminobenzoate-methanesulfonate (i.e., tricaine; Sigma) and immobilized in a scored organ culture dish (BD Falcon) with 1% Low-Melt Agarose (GenePure) in embryo medium. Solidified agarose was then covered with embryo medium containing tricaine and PTU and imaged under a 20× dipping objective using the multipoint timelapse function of an FV1000 confocal microscope (Olympus). Z-stack images were taken every 6–15 minutes over 12–20 hour sessions.

### Transplants

Donor embryos for heterochronic cell transplantation were produced by incrossing *AB wild-type or *cls* heterozygous carriers and injecting zygotes with 1 nL of 10 kD dextran-conjugated rhodamine (Invitrogen) in 0.2 M KCl (Sigma). Donors and *prdm1a^nl3^* mutant hosts were dechorionated with 1 mg/mL Pronase (Sigma) and allowed to develop to the sphere and shield stage, respectively. About 30–50 donor cells were then excised from labeled blastulae with a microaspiration pipette apparatus (WPI) attached to a precision micromanipulator (Siskiyou Instruments) and transferred to the presumptive neural crest field in a shield-stage mutant host. The procedure was performed using an Axioplan2 upright fixed stage microscope (Zeiss).

### Image Acquisition and Processing

Raw images were obtained with an Imager Z1 compound microscope using the AxioVision software package (Zeiss). Raw images from the FV1000 confocal microscope were acquired with FluoView software (Olympus). Post-processing of single-plane and z-stack still images was carried out using ImageJ [32] and Fiji (http://pacific.mpi-cbg.de); when applicable, punctate background was reduced in whole image sets by employing the ImageJ outlier removal filter. Z-projection images were produced using ImageJ, Fiji and FluoRender (Scientific Computing and Imaging Institute, University of Utah). Composite images were produced in Fiji by removing punctate background with the despeckle filter and creating partial z-stack projections for individual channels. Channel projections were then superimposed using the color merge module to preserve anteroposterior and dorsoventral spatial relationships between structures situated at different depths (z-positions). Time-lapse movies were projected, concatenated and formatted in ImageJ. Figures were assembled with Photoshop and Illustrator (Adobe).

### Cell Counts and Statistics

Manual cell counts were carried out on z-stack projection images of the EB placodes using the Point Picker utility in Fiji (http://pacific.mpi-cbg.de). Seven mutant embryos and seven wildtype sibling controls were analyzed for each assay. Mitotic indices were calculated as the number of phospho-histone H3-positive cells that were also Pax2-positive divided by the total number of Pax2-positive cells in the EB placodes. Statistics were generated using the Wilcoxon Signed Rank test in JMP statistical software (SAS Institute).

## Supporting Information

Figure S1 **Glial NC cells are normal in the** ***prdm1a^nl3^*** **mutant.** Lateral view showing that comparable *foxd3* expression is detected by *in situ* hybridization in the region of the otic vesicle (ov) in wildtype (A) and *prdm1a^nl3^* mutant embryo (B) at 48 hpf. Abbreviation: OV = otic vesicle. Scale bar in (A) = 50 µm.
(TIF)

Figure S2 **Endodermal pouches are present in NC-depleted mutants.** (**A–D**) Ventral views showing *nkx2.3* expression as detected by *in situ* hybridization in the head of wildtype (A) and *foxd3* (B), *sox10* (C) and *prdm1a* (D) mutants at 2 dpf. Endodermal pouches expressing *nkx2.3* transcript are visible as bilateral sets of parallel linear expression domains posterior to the eyes (A, brackets). (**E and F**) Lateral view of Zn-5 immunofluorescence (red) marking endodermal pouches at 5 dpf in wildtype (E, arrowheads) and *disc-1* morphant (F). Embryos were stained with DAPI (blue) to visualize nuclei. Scale bar in (A) = 100 µm. Scale bar in (E) = 50 µm.
(TIF)

Figure S3 **Relationship between CB arches and EB ganglia formation in** ***prdm1a^nl3^*** **mutants.** (**A**) Composite lateral view of EB ganglia visualized with *Tg(neuroD:EGFP)^nl1^* transgene (green) alongside cartilaginous CB arches stained for Collagen2a (red) in a 4 dpf wildtype larva. Fluoresence demonstrates spatial association of the first ceratobranchial arch with the glossopharyngeal ganglion and the second through fifth ceratobranchial arches with the first through fourth vagal ganglia, respectively. (**B–E**) Composite lateral views of *prdm1a^nl3^* mutant larvae exhibiting different degrees of CB arch formation. (B) A single, normally-formed first CB ventral to a cluster of EGFP-positive cells that corresponds to the glossopharyngeal ganglion. Note the absence of other small ganglia. (C) Normally-formed first CB arch alongside a dysmorphic second ceratobranchial arch. A fused ganglion structure is visible dorsal to the arches (bracket). (D) Normal formation of first and second CB arches. Distinct glossopharyneal and first vagal ganglia are positioned dorsal to the arch bodies. (E) Normally-formed first CB arch with dysmorphic second arch and presumptive third arch vestige. As in D, distinct glossopharygeal and first vagal ganglia are visible dorsal to arch structures. In addition, a small EGFP-positive cluster of cells can be seen between the arches and ganglia. Abbreviations: G = glossopharyngeal ganglion; V1–V4 = first through fourth vagal ganglia; cb1–cb5 = ceratobranchial arches 1–5. Scale bar = 50 µm.
(TIF)

Figure S4 **Spatial relationship between neurons, glia, and cartilage in the zebrafish head.** (**A**) Lateral view of cranial ganglia in a 5 dpf wildtype larva expressing *Tg(neurod:EGFP)* and stained for Collagen2a (red). The rostral basicranial commisure (asterisk) can be seen positioned dorsally to the cranial ganglia. The Collagen2a-positive branchial arches are located ventrally (arrows). (**B**) Lateral view of cranial ganglia in a 5 dpf wildtype larva as visualized with immunofluoresence against Elavl-3/4 (green). Glial cells are stained with an antibody recognizing MBP (red). Scale bar in (A) = 50 µm.
(TIF)

Movie S1 **Neural crest migrates to the region of the EB placodes prior to ganglion formation.** Timelapse of confocal projections showing a wildtype embryo expressing *TgBAC(neuroD:EGFP)^nl1^* with photoconverted Kaede (red) marking cells in the region of somite 6. Images were acquired every 7.8 minutes for ∼14 hours. By ∼19 hpf, Kaede-positive cells corresponding to migrating NC can be seen streaming ventrally to populate the region proximal to the forming posterior lateral line ganglion (pLL). The first vestiges of the condensing major vagal ganglion (gX) are not seen by EGFP until ∼30 hpf (white arrow). Abbreviations: r6 = rhombomere 6; OP = otic placode; gV = trigeminal ganglion; gAll – anterior lateral line ganglion; gVIII = statoacoustic ganglion; gPll = posterior lateral line ganglion; prim = posterior lateral line primordium; OV = otic vesicle; gX = vagal ganglion. Scale bar = 100 µm.
(MOV)

Movie S2 **Epibranchial ganglion assembly in the wildtype zebrafish.** Timelapse of confocal projections showing a wildtype embryo expressing *TgBAC(neuroD:EGFP)^nl1^* in the region of the otic vesicle beginning at 31 hpf. Images were acquired every 8.4 minutes over ∼15 hours. Exemplars of cells in the vicinity of the glosopharyngeal/vagal placodal field (arrows; 33.52 hpf) can be seen upregulating EGFP and moving dorsally to form the large vagal ganglion. By the end of the acquisition at 45.84 hpf, the facial and glossopharyngeal ganglia have formed and the vagal ganglion is in the process of segmentation. Abbreviations: F = facial ganglion; G = glosspahryngeal ganglion; V = vagal ganglia. Scale bar = 50 µm.
(MOV)

Movie S3 **Epibranchial ganglion assembly in the** ***prdm1a^nl3^*** **mutant.** Timelapse of confocal projections showing a *prdm1a^nl3^* embryo expressing *TgBAC(neuroD:EGFP)^nl1^* in the region of the otic vesicle beginning at 31 hpf. Images were acquired every 8.4 minutes over ∼15 hours. As in the wild type, cells forming the major vagal ganglion upregulate EGFP as assembly commences. However, by 46.26 hpf, only the facial and large vagal ganglia can be identifiied. The major vagal ganglion is elongated compared to wildtype cohorts and shows no signs of segmentation. In addition, a small group of cells (asterisk; 46.26 hpf) is visible ventral to the otic vesicle in the location occupied by the glossopharyngeal and nascent minor vagal ganglia in wild type. Abbreviations: F = facial ganglion; V = vagal ganglia. Scale bar = 50 µm.
(MOV)

Movie S4 **Late-stage epibranchial ganglion morphology is stable in the wildtype zebrafish.** Timelapse of confocal projections showing the region of the otic vesicle in a wildtype embryo expressing *TgBAC(NeuroD:EGFP)nl1* between 3 and 4 dpf. The ganglia continue to condense and undergo segmentation during this period, but the overall structure remains largely stable. Images were acquired every 15 minutes over a period of 17.25 hours. Scale bar = 50 µm.
(MOV)

Movie S5 **Epibranchial ganglion morphology in the sox10 mutant.** Timelapse of confocal projections showing a *sox10* mutant embryo expressing *TgBAC(NeuroD:EGFP)^nl1^* in the region of the otic vesicle between 3 and 4 dpf. Though the ganglia have undergone segmentation, a number of cells can be seen dissociating from the ganglia (arrows). Images were acquired every 15 minutes over a period of 17.25 hours. Scale bar = 50 µm.
(MOV)

Table S1 (DOCX)
