# Supplementary figures and images for: Chondrogenic and Gliogenic Subpopulations of Neural Crest Play Distinct Roles during the Assembly of Epibranchial Ganglia
Source: PLoS One. 2011 Sep 9;6(9):e24443. doi: 10.1371/journal.pone.0024443 (PMC3170370; doi:10.1371/journal.pone.0024443)

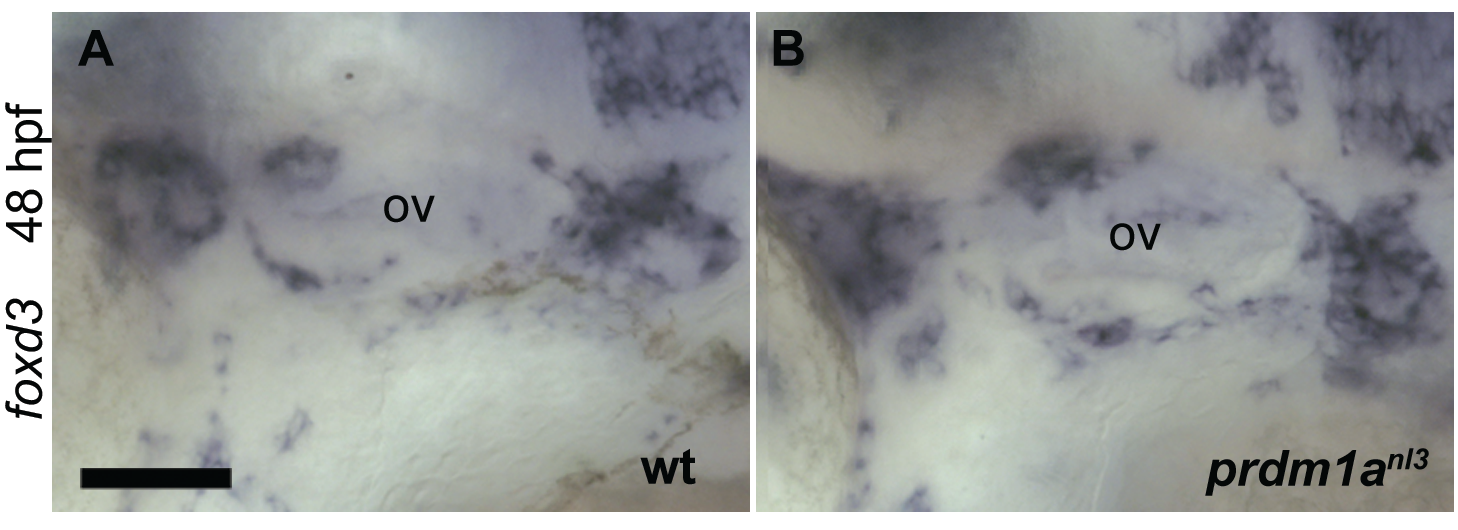

Supplement: Figure S1 — Glial NC cells are normal in the prdm1anl3 mutant. Lateral view showing that comparable foxd3 expression is detected by in situ hybridization in the region of the otic vesicle (ov) in wildtype (A) and prdm1anl3 mutant embryo (B) at 48 hpf. Abbreviation: OV = otic vesicle. Scale bar in (A) = 50 µm. (TIF) [file pone.0024443.s001.tif]

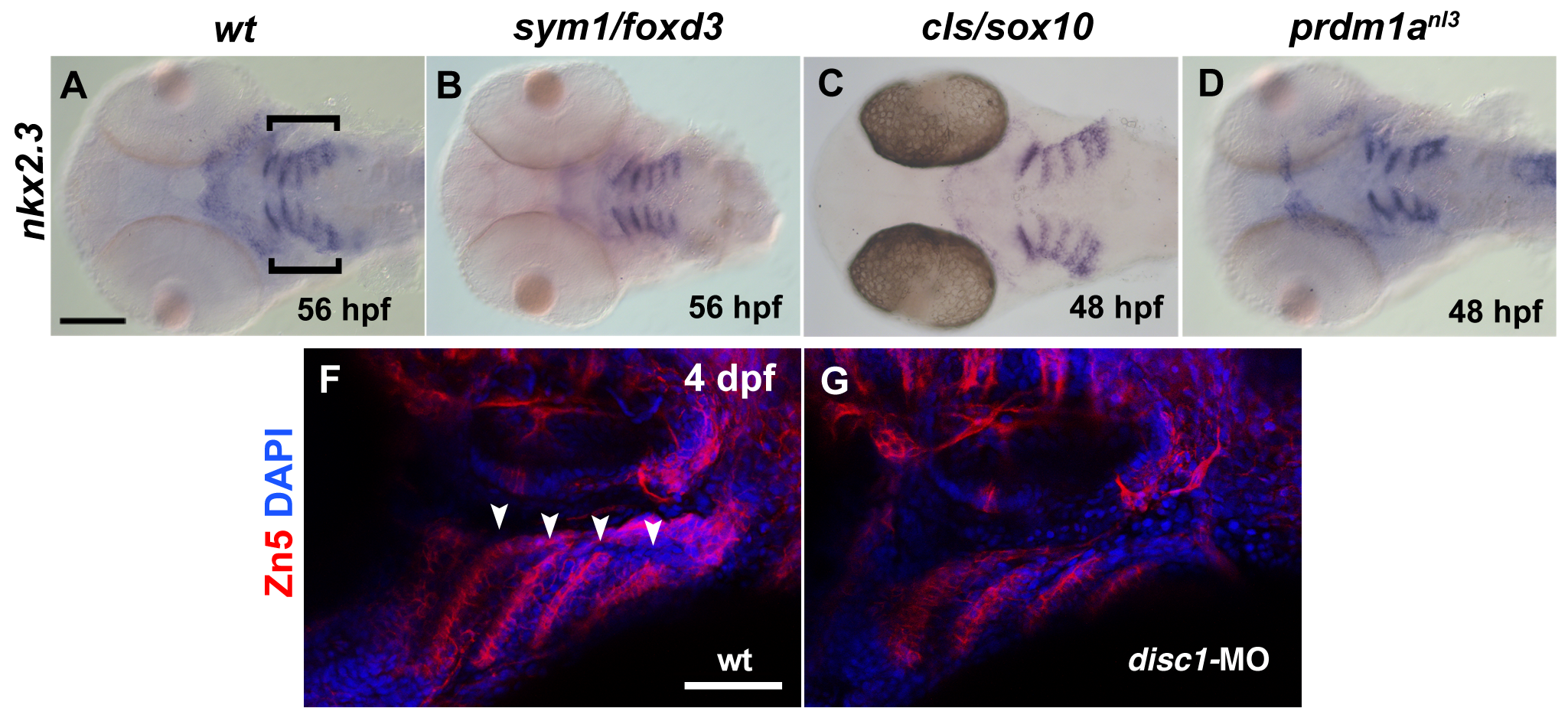

Supplement: Figure S2 — Endodermal pouches are present in NC-depleted mutants. (A–D) Ventral views showing nkx2.3 expression as detected by in situ hybridization in the head of wildtype (A) and foxd3 (B), sox10 (C) and prdm1a (D) mutants at 2 dpf. Endodermal pouches expressing nkx2.3 transcript are visible as bilateral sets of parallel linear expression domains posterior to the eyes (A, brackets). (E and F) Lateral view of Zn-5 immunofluorescence (red) marking endodermal pouches at 5 dpf in wildtype (E, arrowheads) and disc-1 morphant (F). Embryos were stained with DAPI (blue) to visualize nuclei. Scale bar in (A) = 100 µm. Scale bar in (E) = 50 µm. (TIF) [file pone.0024443.s002.tif]

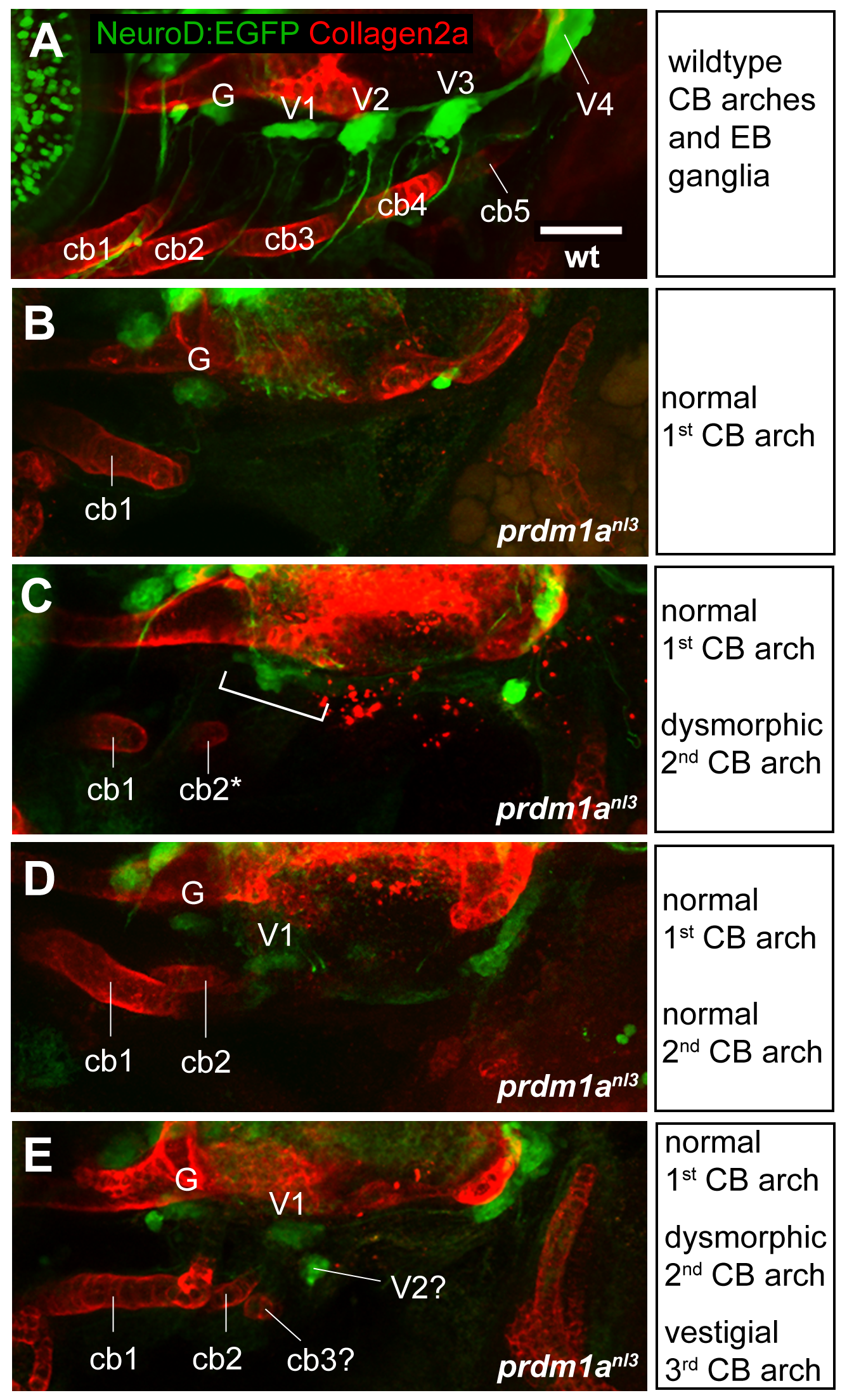

Supplement: Figure S3 — Relationship between CB arches and EB ganglia formation in prdm1anl3 mutants. (A) Composite lateral view of EB ganglia visualized with Tg(neuroD:EGFP)nl1 transgene (green) alongside cartilaginous CB arches stained for Collagen2a (red) in a 4 dpf wildtype larva. Fluoresence demonstrates spatial association of the first ceratobranchial arch with the glossopharyngeal ganglion and the second through fifth ceratobranchial arches with the first through fourth vagal ganglia, respectively. (B–E) Composite lateral views of prdm1anl3 mutant larvae exhibiting different degrees of CB arch formation. (B) A single, normally-formed first CB ventral to a cluster of EGFP-positive cells that corresponds to the glossopharyngeal ganglion. Note the absence of other small ganglia. (C) Normally-formed first CB arch alongside a dysmorphic second ceratobranchial arch. A fused ganglion structure is visible dorsal to the arches (bracket). (D) Normal formation of first and second CB arches. Distinct glossopharyneal and first vagal ganglia are positioned dorsal to the arch bodies. (E) Normally-formed first CB arch with dysmorphic second arch and presumptive third arch vestige. As in D, distinct glossopharygeal and first vagal ganglia are visible dorsal to arch structures. In addition, a small EGFP-positive cluster of cells can be seen between the arches and ganglia. Abbreviations: G = glossopharyngeal ganglion; V1–V4 = first through fourth vagal ganglia; cb1–cb5 = ceratobranchial arches 1–5. Scale bar = 50 µm. (TIF) [file pone.0024443.s003.tif]

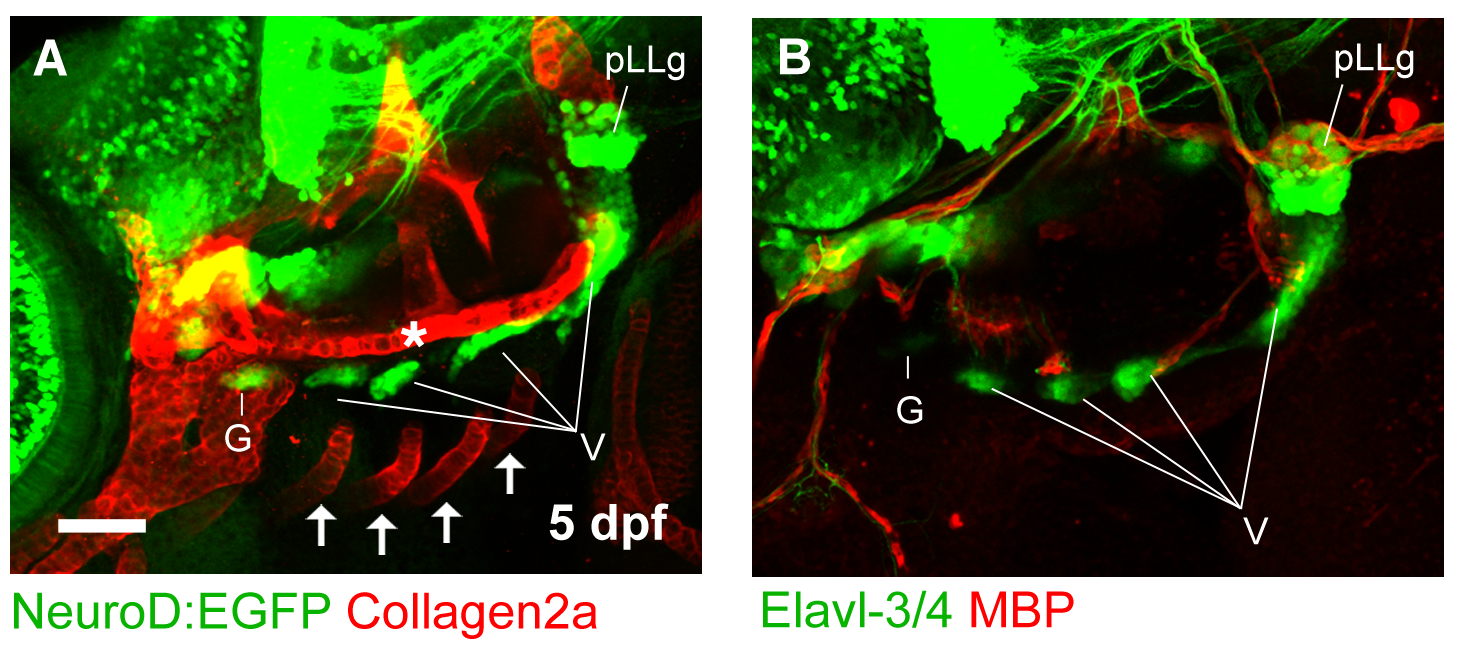

Supplement: Figure S4 — Spatial relationship between neurons, glia, and cartilage in the zebrafish head. (A) Lateral view of cranial ganglia in a 5 dpf wildtype larva expressing Tg(neurod:EGFP) and stained for Collagen2a (red). The rostral basicranial commisure (asterisk) can be seen positioned dorsally to the cranial ganglia. The Collagen2a-positive branchial arches are located ventrally (arrows). (B) Lateral view of cranial ganglia in a 5 dpf wildtype larva as visualized with immunofluoresence against Elavl-3/4 (green). Glial cells are stained with an antibody recognizing MBP (red). Scale bar in (A) = 50 µm. (TIF) [file pone.0024443.s004.tif]
