# Supplementary material for: Chondrogenic and Gliogenic Subpopulations of Neural Crest Play Distinct Roles during the Assembly of Epibranchial Ganglia
Source: PLoS One. 2011 Sep 9;6(9):e24443. doi: 10.1371/journal.pone.0024443 (PMC3170370; doi:10.1371/journal.pone.0024443)
Supplement: Table S1 — (DOCX) [file pone.0024443.s010.docx]

**Table 1:** Presence of 1^st^ minor vagal ganglion versus 2^nd^ ceratobranchial arch formation

|  | Absent 2^nd^ Ceratobranchial (7/11) | Dysmorphic 2^nd^ Ceratobranchial (3/11) | Normal 2^nd^ Ceratobranchial (1/11) |
| --- | --- | --- | --- |
| Number of 1^st^ Minor Vagal Ganglia Formed | 1 | 2^‡, †^ | 1^‡^ |

‡ - one fusion with glossopharyngeal ganglion

† - one dysmorphic
